# Early detection and intervention for young children with early developmental disabilities in Western Uganda: a mixed-methods evaluation

**DOI:** 10.1186/s12887-022-03184-7

**Published:** 2022-03-26

**Authors:** S. Sadoo, R. Nalugya, R. Lassman, M. Kohli-Lynch, G. Chariot, H. G. Davies, E. Katuutu, M. Clee, J. Seeley, E. L. Webb, R. Mutoni Vedastine, F. Beckerlegge, C. J. Tann

**Affiliations:** 1 London School of Hygiene and Tropical Medicine, Keppel Street, London, UK; 2 Neonatal Medicine, University College London Hospitals NHS Trust, London, UK; 3 Spina bifida and Hydrocephalus association of Uganda, Kampala, Uganda; 4 MRC/UVRI & LSHTM Uganda Research Unit, Entebbe, Uganda; 5 Kyaninga Child Development Centre, Fort Portal, Kabarole Uganda; 6 Centre for Child and Adolescent Health, University of Bristol, Bristol, UK; 7 St George’s University London, London, UK; 8 Fort Portal Regional Referral Hospital, Fort Portal, Kabarole Uganda

**Keywords:** Developmental disability, Uganda, Early detection, Early intervention

## Abstract

**Background:**

Early support for children with developmental disabilities is crucial but frequently unavailable in low-resource settings. We conducted a mixed-methods evaluation to assess the feasibility, acceptability, and impact of a programme of early detection and intervention for young children with developmental disabilities in Western Uganda.

**Methods:**

Early child development training for healthcare workers (HCWs) was implemented in three rural districts, and attendance was tracked. HCW knowledge and confidence were assessed pre-/post-intervention, and referral numbers tracked to evaluate impact. Facilitators were trained and mentored to deliver a participatory, group, early intervention programme (EIP) for young children with developmental disabilities and their families. Facilitators were tracked as they were identified, trained, and delivered the intervention, and attendance of families was tracked. Pre−/post-intervention assessments evaluated changes in family quality of life (PedsQL 2.0, Family Impact Module), and child nutritional outcomes. Focus group discussions with stakeholders also assessed feasibility, acceptability and impact.

**Results:**

Overall, 93 HCWs from 45 healthcare facilities received training. In the pre−/post-evaluation, median knowledge and confidence scores increased significantly (from 4.0 to 7.0 and from 2.7 to 4.7, respectively (*p* < 0.001)). HCWs reported feeling empowered to refer and offer care for families with a young child with disability. Referral rates increased significantly from 148 to 251 per annum (70%; *p* = 0.03). Eleven EIP facilitators were trained, and all delivered the intervention; 84 families were enrolled, of which 78% attended at least 6 out of 10 modules. Amongst those with paired pre−/post-intervention data (*n* = 48), total family quality of life scores increased significantly (21%, *p* < 0.001). Improvements were seen across all domains of quality of life, with the largest impacts on emotional functioning and social functioning (*p* < 0.001). The programme was acceptable to caregivers and facilitators. Caregivers reported improved knowledge, family relationships, hope, emotional wellbeing, and reduced self-stigma.

**Conclusions:**

A programme of early detection and intervention for children with early developmental disabilities and their families was feasible and acceptable in a rural community-based Ugandan setting. HCW training positively impacted knowledge, confidence, attitudes, and referral rates. Families enrolled to the EIP reported significant improvements in quality of life. Important programmatic barriers identified included geographical spread, poverty, gender inequality, and stigma.

## Introduction

Whilst significant progress has been made in reducing child mortality in low- and middle-income countries (LMICs) over recent decades, the number of children with developmental disabilities has remained unchanged [1]. An estimated 52.9 million children under-five currently live with developmental disabilities worldwide, 95% of whom reside in LMICs [1]. Children with developmental disabilities face substantial social, emotional and financial impacts particularly in LMICs, where frequently poverty levels are higher, access to services limited, and social stigma more evident [2].

Early intervention for children with developmental disabilities is increasingly recognised as a priority on the global agenda. The UN Sustainable Development Goal target 4.2 aims for all children to have access to quality early child development and care [3], and the Global Strategy for Women’s, Children’s and Adolescents’ Health (2016-2030) advocates in its three core pillars that children should not only ‘survive’, but ‘thrive’ [4]. The WHO/UNICEF/World Bank Nurturing Care Framework endorses responsive caregiving and early learning [5], and the UN Convention on the Rights of the Child and UN Convention of the Rights of Persons with Disabilities state that governments should provide early years services that are inclusive of and available to all children [6]. Yet, children with developmental disabilities are frequently unable to access services [7].

Early childhood interventions have the potential to improve family quality of life, and reduce functional impairment in high-risk newborns either directly or indirectly, for example through enhancing the care-giving environment [8–10]. However, delayed identification and referral is common in LMICs which can worsen outcomes. For example, feeding difficulties can lead to severe malnutrition, and motor impairments can cause contractures and deformities without adequate therapy [6]. The evidence base around early intervention is growing in LMICs with several studies demonstrating promising impacts on young children and their families [11–14]. However, gaps in the literature persist. Whilst early identification is recognised as a crucial element of early intervention, there is no consensus on how to detect at risk infants in LMICs. Further, data are lacking on recruitment, training and retention of HCWs needed to identify infants with suspected developmental delay/disability who warrant referral for specialist assessment.

### Aims

We aimed to evaluate a programme of early detection and intervention for young children (age 0-3 years) with early developmental disabilities in western Uganda. Conducting secondary analysis of anonymised, de-identified mixed-methods data, we aimed to evaluate:

1. *Feasibility, acceptability and impact* of a healthcare worker (HCW) training programme to detect and refer young children with early developmental disabilities.
2. *Feasibility, acceptability and impact* of the community-based, participatory, group Baby Ubuntu programme of early care and support for young children with developmental disabilities and their families.

## Methods

### Setting

**Fig. 1:**
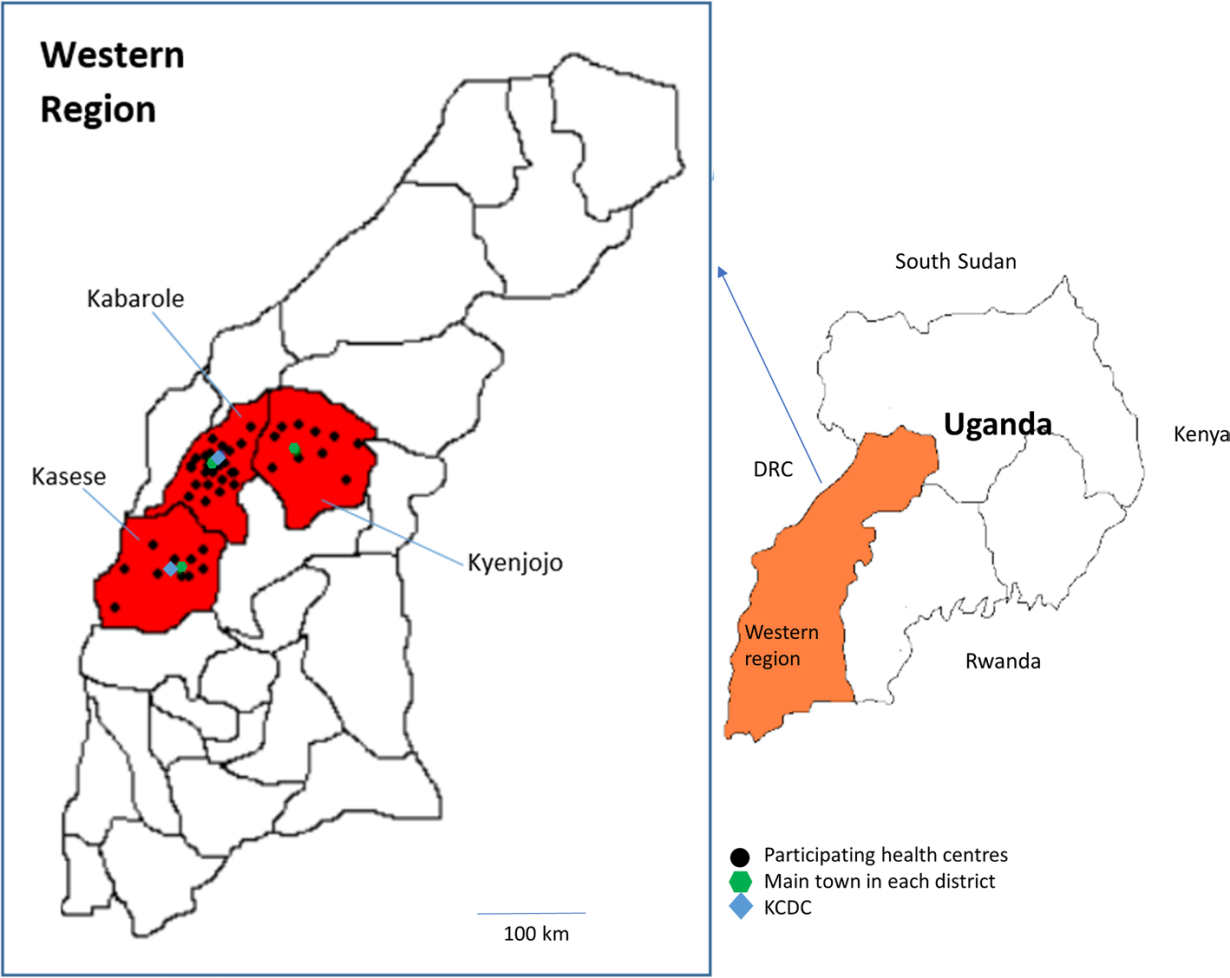
Map of Kyenjojo, Kabarole and Kasese districts in the Western region of Uganda. All distances and locations are approximate. Created by authors in Windows XP MS Paint, adapted from Map of Uganda published by OCHA (2006) accessed 21st July 2021: https://reliefweb.int/map/uganda/map-uganda-including-new-districts-region-jul-2006

Uganda is a low-income country in East Africa, ranking 176 out of 193 countries worldwide for GDP per capita [15]. It is estimated that 3.4% of all Ugandan children aged 2-4 years and 7.5% aged 5-17 years are living with a disability [16], and only 10% of these children have access to rehabilitative services [17]. The districts of Kyenjojo, Kabarole, and Kasese lie in the Western region, encompassing a total area of 7000 km^2^ and a population of 1.6 million, of which 77% live in a rural area (Fig. 1). The main town in each district are the only urban areas, the rest of the region being largely rural. There are two child development centres in the Western region (Kyaninga Child Development Centre; KCDC); one located in the town of Fort Portal (Kabarole), and the other located in Kasese town (Kasese). KCDC provides rehabilitation services for children with disabilities, offering physiotherapy, occupational therapy, speech and language therapy, orthopaedic therapy, special needs education support, and adaptive equipment. KCDC receives referrals from government health centres and provides outreach clinics across the region.

### Healthcare worker training for early detection of developmental disability

A structured early child development (ECD) training programme for HCWs was developed and piloted in December 2017, with the aim of promoting early detection of young children with developmental disability. Content included developmental milestones, ‘red-flag’ signs for developmental delay and disability, communicating with parents, and principles of early intervention. Training included presentations, videos, and group discussions, and was as participatory as was feasible. Educational resources such as developmental milestone charts were provided for use at participating HCWs’ health facilities to aid assessment. HCWs who had regular contact with children under 2 years from health centres across the 3 districts were invited to training delivered by KCDC therapists. A 1 day face-to-face training session was followed by a 1 day ‘refresher’ training session 6 months later, to consolidate learning and track knowledge retention. Ongoing mentorship was provided through social media application and KCDC outreach clinics, to maintain fidelity of the programme.

#### Data collection for early detection

##### Quantitative data

Data were recorded on standardised data collection forms, comprising the number of health facilities invited, and the numbers and cadres of HCWs attending initial and refresher training. HCWs completed pre- and post-training assessments on ECD knowledge, confidence and skills immediately before and after the initial training session, and this was repeated immediately prior to the refresher training 6 months later to assess attrition. Self-assessment of confidence in i) understanding of cerebral palsy, ii) assessment of developmental milestones and delay at 6-24 months, and iii) parental communication, was scored on a 5-point Likert scales (1 equating to ‘not at all confident’ and 5 to ‘very confident’). Assessment of knowledge and skills comprised four free-text questions with a maximum score of 8. These questions covered understanding and aetiology of cerebral palsy and knowledge of age-appropriate developmental milestones. Assessments were scored by KCDC therapists. Monthly referrals received by KCDC for young children with suspected developmental disabilities were recorded.

Outcome measures for the feasibility and acceptability of HCW training to detect and refer young children with suspected developmental disabilities, included the number of health centres invited to training, number of HCWs who attended the initial training, and number of HCWs who attended the subsequent refresher training. Impact of HCW training was evaluated by pre-post changes in knowledge & confidence scores, and changes in referral rates for children with disability over time.

##### Qualitative data

Data were collected through focus group discussions (FGDs) with HCWs conducted by an experienced social scientist, using a semi-structured guide to explore perceptions of the ECD training programme and impact on confidence levels, attitudes and practice. Discussions recorded at the second stakeholders meeting held following completion of the programme were also included in the qualitative analysis.

### Early intervention Programme

The “Baby Ubuntu” (formerly the “ABAaNA”) early intervention programme (EIP) is a community-based, modular, participatory, group programme for children and their caregivers. Details about the development and piloting of the programme have been published previously [18, 19].

It is designed to increase parental knowledge, skills and confidence in caring for a child with disability [18–21]. It aims to allow children to fulfil their developmental potential, optimise health, and improve quality of life of both the child and family. Over 10 modules, families learn about disability, positioning and carrying, feeding, mobilising, communication, play, everyday activities, and experiences in the local community [18]. The programme is facilitated by a trained ‘expert parent’ facilitator, themselves a parent of a child with disability, and sessions are held in the community to enhance accessibility, including one home visit.

‘Expert-parent’ facilitators were identified by therapists and invited to training based on their confidence, willingness, and capability to learn and teach the EIP manual. They underwent 5 days of core training, followed by regular supervision and mentoring in person and via telephone and a social media application, in order to provide support in running groups and maintain fidelity to the EIP. Established across Kabarole and Kasese districts, families were assigned to EIP groups according to locality, each comprising 6-10 families of children who were clinically assessed to have early developmental disability by trained therapists. Individual module sessions were delivered every 1-2 weeks lasting 2-3 h, depending on the preferences and availabilities of the group; the entire programme was delivered over 6 months.

#### Data collection for early intervention

##### Quantitative data

Data was recorded on standardised data collection forms on the number of facilitators identified, trained, and delivering the EIP. Outcome measures recorded to assess feasibility and acceptability of the EIP included; the number of EIP facilitators invited to training, number of invited EIP facilitators completing training; number of trained facilitators delivering the programme; number of families enrolled to the EIP; number of families with satisfactory attendance (defined as completion of ≥6 modules).

Community-level EIP impact was measured by the number of EIP groups run. Impact on child and family quality of life was evaluated using the Pediatric Quality of Life Inventory; Family Impact module 2.0 (PedsQL) pre- and post- intervention, within 1 month prior to commencing module 1, and immediately after the final module, respectively [21]. The PedsQL is a scored, validated research tool successfully used in pilot work in Uganda, which was translated and administered in the local language (Rutooro) and conducted by trained therapists and EIP facilitators [22]. It comprises 36 items assessing caregivers’ self-reported physical, emotional, social, and cognitive functioning, communication, worry, daily activities, and family relationships, scored on a 0-4 Likert scale and linearly transformed (scale of 0-100) with higher scores indicating a higher quality of life [19]. Mid-upper arm circumference (MUAC) assessed child nutritional status pre- and post- intervention. Moderate-severe acute malnutrition was defined as a MUAC of < 12.5 cm (severe acute malnutrition being < 11.5 cm) [23].

##### Qualitative data

Data were recorded through FGDs conducted with caregivers receiving the EIP (one group) and EIP facilitators (one group). FGDs facilitated by an experienced social scientist using a semi-structured guide, explored perceptions of the ECD training programme and the impact on the child, confidence level of parents, and level of inclusion in family and community life. FGDs were conducted in the local language and translated into English. Discussions from the second stakeholders meeting held following completion of the programme were also included in the qualitative analysis.

### Data analysis

Secondary analysis of anonymised, de-identified and unlinked programmatic monitoring and evaluation data was conducted.

#### Quantitative data

For HCW training, descriptive data on reach were summarised to assess feasibility and acceptability. HCW knowledge and confidence scores were summarised using median and interquartile range (IQR) at each time point, with Wilcoxon signed-rank tests used to assess whether median scores differed between pre- and post-initial training (to assess impact of the initial training), and between post-initial and pre-refresher training (to assess whether any improvements were sustained). Numbers of referrals by month were plotted with an automated trend line, and the percentage improvement calculated in comparison to the previous year.

Descriptive data on EIP facilitator identification, completion of training and delivery of the EIP including number of groups, number of families enrolled and attendance for each module, were used to assess feasibility and acceptability. Early evidence of impact was evaluated using PedsQL total scores and sub-scores pre-post intervention. Median pre- and post-scores, and median differences were calculated for total and domain scores and compared using Wilcoxon signed-rank tests to allow for paired data. Impact on child nutrition was measured using MUAC scores, comparing pre- and post-intervention using paired t-tests for continuous outcomes and McNemar’s test for binary outcomes.

#### Qualitative data

A thematic framework approach guided the qualitative data analysis, based on data and themes derived from FGDs. The thematic analysis involved reading the narratives, developing a coding framework, and sorting the coded data into summaries and meaningful themes. The social scientist discussed themes with team members at the MRC/UVRI & LSHTM Uganda Research Unit for quality control.

### Participant and public involvement

Both the ECD training and early intervention programmes were developed directly from engagement with HCWs, EIP facilitators and caregivers of children with disability [16]. An initial stakeholders meeting held prior to the start of the intervention was attended by 49 representatives from local healthcare facilities, non-governmental organisations, local government offices and caregivers of children with disabilities; feedback obtained helped to further develop and refine the intervention. Findings from the programme evaluation were communicated to participants and the wider community including through a second stakeholders meeting, attended by 50 representatives from the aforementioned groups, held in Fort Portal following programme completion.

## Results

### Healthcare worker training to detect and refer young children with early developmental disability

**Fig. 2:**
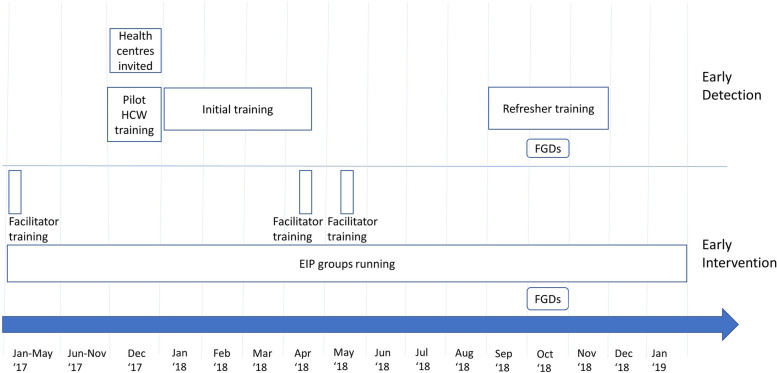
Timeline for healthcare worker training in Early Detection of developmental disability, and Early Intervention facilitator training

Initial ECD HCW training in Kabarole, Kasese and Kyenjojo districts occurred between January and April 2018, and refresher training 6 months later between September and November 2018 (Fig. 2). For the qualitative evaluation, two FGDs were conducted with HCWs in October 2018; one group of 9 HCWs, one group of 5 HCWs, with each group comprising representatives from all three districts.

#### Feasibility and acceptability of HCW training

**Fig. 3:**
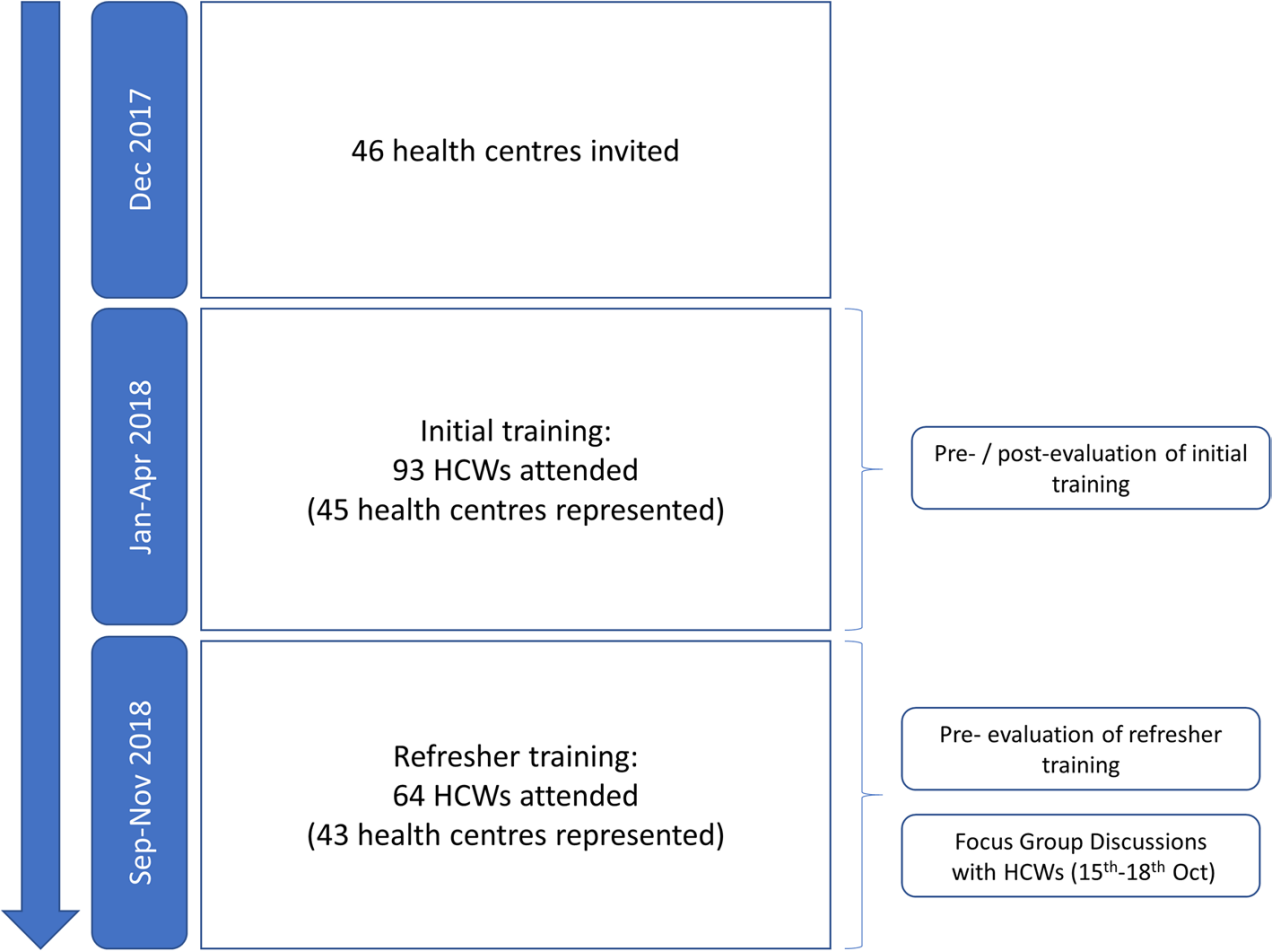
Flow of participants for healthcare worker (HCW) training

Of 46 health centres invited, 45 (99%) were represented in the initial training, and 43 (93%) in the refresher training (Fig. 3). In total 93 HCWs attended initial training, 69% (64) of whom subsequently attended refresher training (Fig. 3). Basic demographics of HCWs are presented in Table 1.

**Table 1:** Basic demographics and clinical characteristics of healthcare workers who attended early child development training (*n* = 93)

	Initial training	Refresher training
Sex	*N* = 93	*N* = 64
	Female 69 (74%)	Female 48 (75%)
	Male 24 (26%)	Male 16 (25%)
Role	*N* = 93	*N* = 64
	Clinical officer 4 (4%)	Clinical officer 2 (3%)
	Nurse 37 (40%)	Nurse 25 (39%)
	Midwife 25 (27%)	Midwife 15 (23%)
	Nursing officer 17 (18%)	Nursing officer 16 (25%)
	Nursing assistant 7 (8%)	Nursing assistant 4 (6%)
	Medical records assistant 3 (3%)	Medical records assistant 2 (3%)
Health centres represented	*N* = 45	*N* = 43
	Kabarole 24 (53%)	Kabarole 22 (51%)
	Kasese 10 (22%)	Kasese 10 (23%)
	Kyenjojo 11 (24%)	Kyenjojo 11 (26%)

Qualitative findings supported training feasibility and acceptability. Training content was identified as appropriate for participants’ needs, and learning materials were engaging and increased participants’ motivation to learn. Multi-media resources (particularly video clips) and sharing of experiences facilitated learning and promoted behaviour change. Barriers included the need for system strengthening with a schedule of refresher training for sustainability due to frequent transfers and high turnover of staff. One HCW said *“I never had hope in these children, I used to think they were already wasted children and I couldn’t care much, but now I understand that they can improve, and I also give to the mothers hope.”*

#### Impact of HCW training on knowledge, confidence and practice

**Fig. 4:**
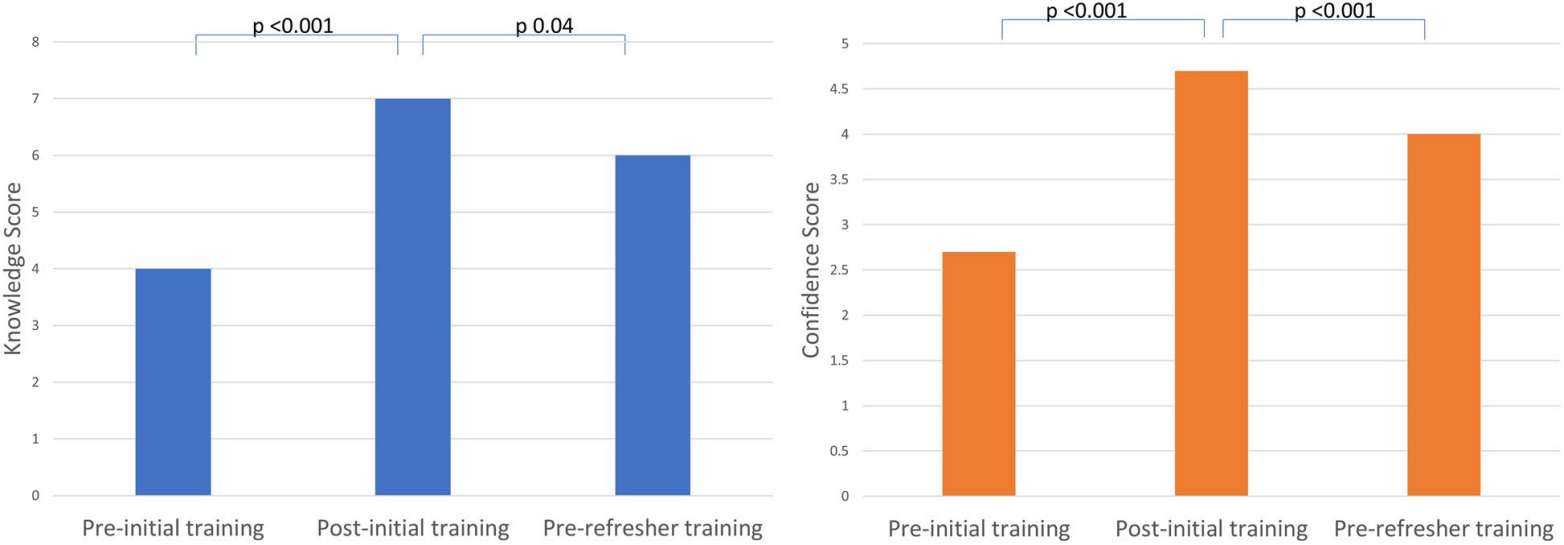
Healthcare worker knowledge and confidence scores; pre-initial training, post-initial training, and pre-refresher training

Scores indicating knowledge and confidence of HCWs were obtained immediately pre- and post- initial training. Before training, the median knowledge score was 4.0 (IQR 3 - 5), and confidence score 2.7 (IQR 2 - 4), increasing significantly following training to 7.0 & 4.7 respectively (both *p* < 0.001) (Fig. 4). At the pre-refresher training assessment, of the 64 attendees, median scores significantly decreased compared to post-initial training, to 6.0 for knowledge and 4.0 for confidence (*p* = 0.04 and < 0.001, respectively) but remained above pre-initial training (baseline) levels.

**Fig. 5:**
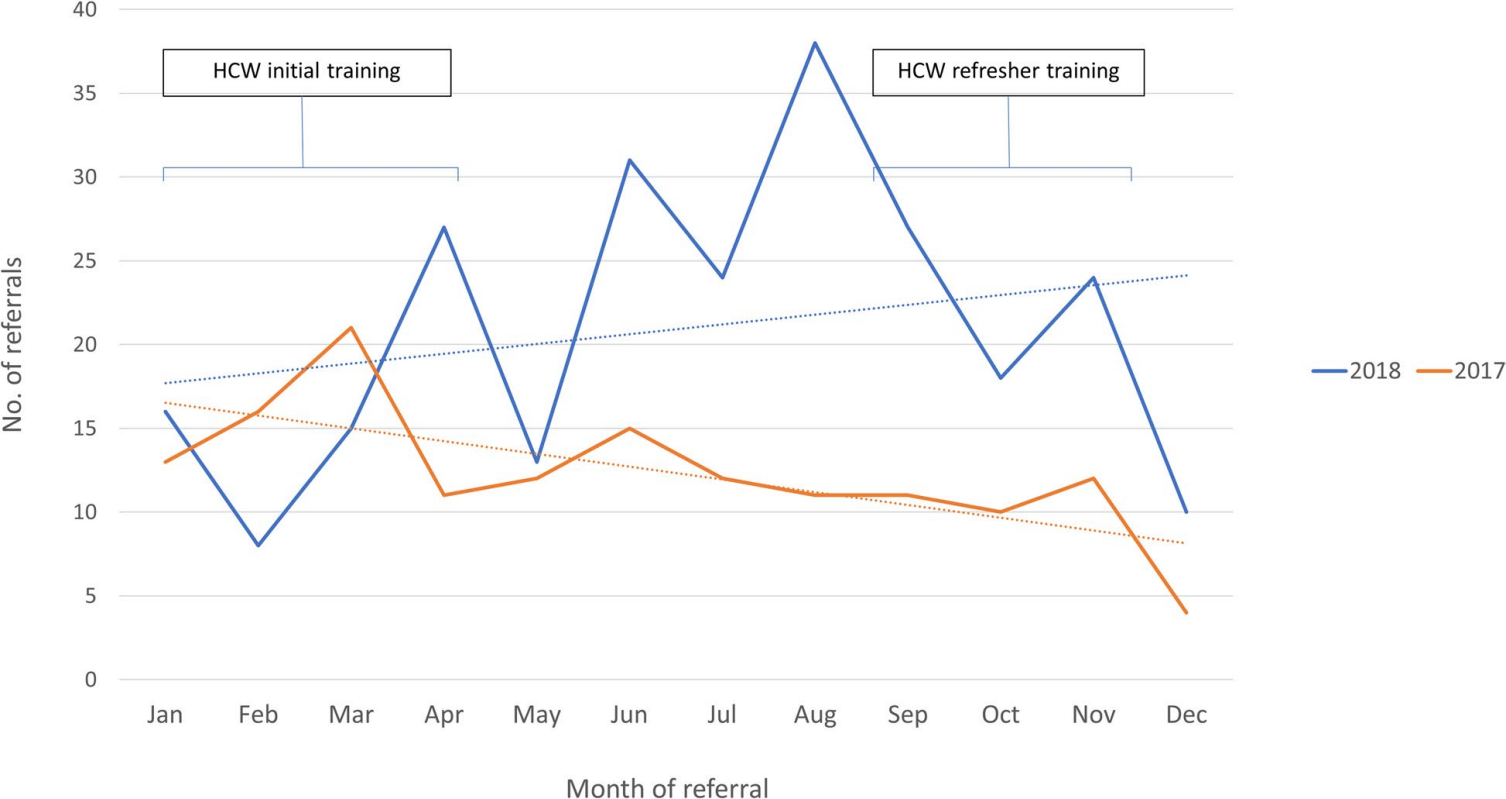
Total number of referrals to KCDC for children under 2 years with suspected developmental disability, by month

The number of appropriate referrals increased significantly by 70% (148 per annum in 2017 to 251 per annum in 2018; *p* = 0.03) (Fig. 5). This equated to an increase in the average monthly referrals from 12.3 per month to 20.9 per month. All health centres were invited to join a social media group facilitated by therapists at the referral centre; 40 out of 45 health centres (89%) had at least one representative HCW in the group.

The information collected through FGDs mirrored the findings in the quantitative data. HCWs reported improved practices and attitudes as a result of training. They explained that improved communication skills positively influenced their relationships with caregivers, and created a supportive environment for children with disabilities in the health facility. Several said that the intervention transformed their behaviours and attitudes towards children with disabilities because of a clearer understanding of the causes, how to identify, assess and refer a child with disability, and manage them both in the hospital and home setting. This new knowledge gave them greater confidence to refer children with developmental disability. For example, one HCW stated that the training was *“an unforgettable experience because she never knew children with disabilities could make it in life and be able to participate”,* after viewing shared video clips of children with cerebral palsy thriving. However, HCWs also expressed concern about increasing numbers of children with disability and gaps in service and skills at health centres to identify children and offer care and support to families.

HCWs mentioned increased access to information concerning hospital care for children with developmental disability. They reported previous lack of guidance, references and information on the disability care pathway and management within the hospital setting, and this affected the delivery of health care to children with growth and development challenges within the area. HCWs reported that the programme provided additional information for example reference books/charts, and a clear map of services that caregivers could access. This led to integration of management of developmental disability within health facilities and improved community and home based care. They mentioned however, that educational materials were limited and some health facilities did not have the electricity and technology to disseminate the information to patients using the video clips within hospitals, and that some training materials were not provided in the local language.

### Evaluating the early intervention programme for young children with developmental disability and their family

#### Feasibility & acceptability of the early intervention Programme

**Fig. 6:**
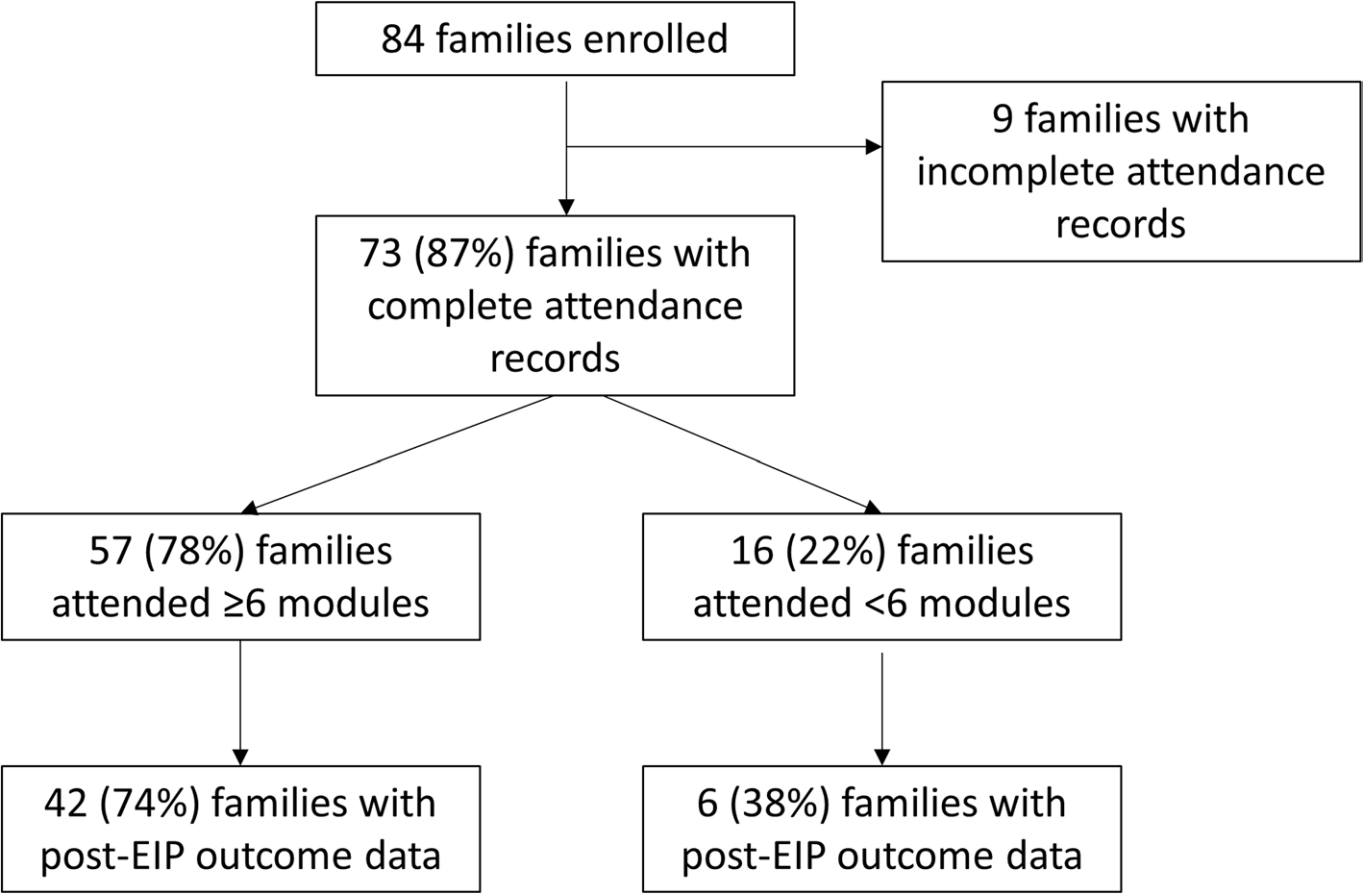
Flow of participants for the early intervention programme

Three 5-day training courses were held in January 2017, April 2018 and May 2018 in Kabarole and Kasese districts, in which 11 parents were trained to become EIP facilitators (Fig. 2). Between January 2017 and January 2019, the EIP was rolled out across all three districts. For the qualitative evaluation, in October 2018 one FGD was conducted with 6 caregivers, and one FGD with 8 EIP facilitators, each FGD comprising participants from all three districts (Fig. 2). Of the 13 parents who were invited to be trained as EIP facilitators, 12 completed training (7 mothers, 5 fathers) and all 12 parents subsequently delivered the programme. Of the 84 enrolled families, 73 had complete attendance records (Fig. 6). Of these, 57 (78%) had satisfactory attendance (≥6 modules attended). Only 4 fathers attended EIP sessions as caregivers.

Our findings from the FGDs and second stakeholder meeting showed that caregivers found participation in the EIP feasible and acceptable, although some barriers were identified. Many participants commented that the venues were desirable in terms of location and access. For the groups that used KCDC as a venue, caregivers reflected that as they had previously attended KCDC for therapies, the venue was a familiar setting for them and their children and therefore attracted them to the EIP. However, several participants mentioned that other community and health facility-based venues were not ideal as, due to the wide geographical spread of participants, some participants found venues more difficult to access than others. In addition, participants commented that some venues lacked space or comfortable seating. Limited finances was cited as a major barrier to attendance, with female caregivers reporting difficulties both in raising transport money to attend sessions and in persuading their husbands to provide the transport money. Female caregivers highlighted lack of support from their partners as another barrier to attendance. One mother commented *“all other family members do care but the man doesn’t... some men don’t care because the children have disabilities, and they think it is a wastage of time.*” Participants reported that despite these barriers of poverty, gender inequality and wide geographical spread, plus others including lack of available transport, poor health of the child, and bad weather, they were encouraged to attend by the warm reception they received from EIP facilitators.

Caregivers and EIP facilitators gave positive feedback on the EIP, finding the content and format relevant and acceptable. Caregivers reported that having similar characteristics to others in their group, for example male gender for fathers, and being in a group setting, reduced anxiety around participating in sessions enhancing acceptability of the EIP for them. Most caregivers and EIP facilitators found the EIP content to be relevant to their child and that it answered many questions they held regarding their child’s condition, care and development. Caregivers commented that EIP facilitators being parents of children with disabilities and sharing their own experiences, was an important part of the EIP. Several participants highlighted the value of learning from other caregivers in how they support their children’s health and development. One caregiver commented “*...fellow mothers give us hope that our children will improve and this kept us strong.”*

#### Impact of the early intervention Programme

A total of nine EIP groups were run across the three districts. Pre-post EIP outcome data on family quality of life and nutrition scores were available for 48 (57%) families, of which 42 (88%) had satisfactory attendance. Baseline and clinical characteristics of participants are presented in Table 2.

**Table 2:** Basic demographics and clinical characteristics of Early Intervention Programme participants with pre-post outcome data (*n* = 48)

Median age at assessment [IQR]	16.2 [IQR 10.0, 24.8] months, range 4.5-54.0 months
Sex distribution (%)	36 (75%) male, 12 (25%) female
Developmental disability diagnosis	46
Cerebral Palsy	44
Spastic unilateral (hemiplegia)	4
Spastic bilateral (quadriplegia)	17
Spastic bilateral (diplegia)	2
Choreo-athetoid	12
Hypotonic	9
Global Developmental Delay	1
Other developmental disability	1
Median attendance at EIP [IQR]	7 modules [6-9]

*IQR* Interquartile range

**Fig. 7:**
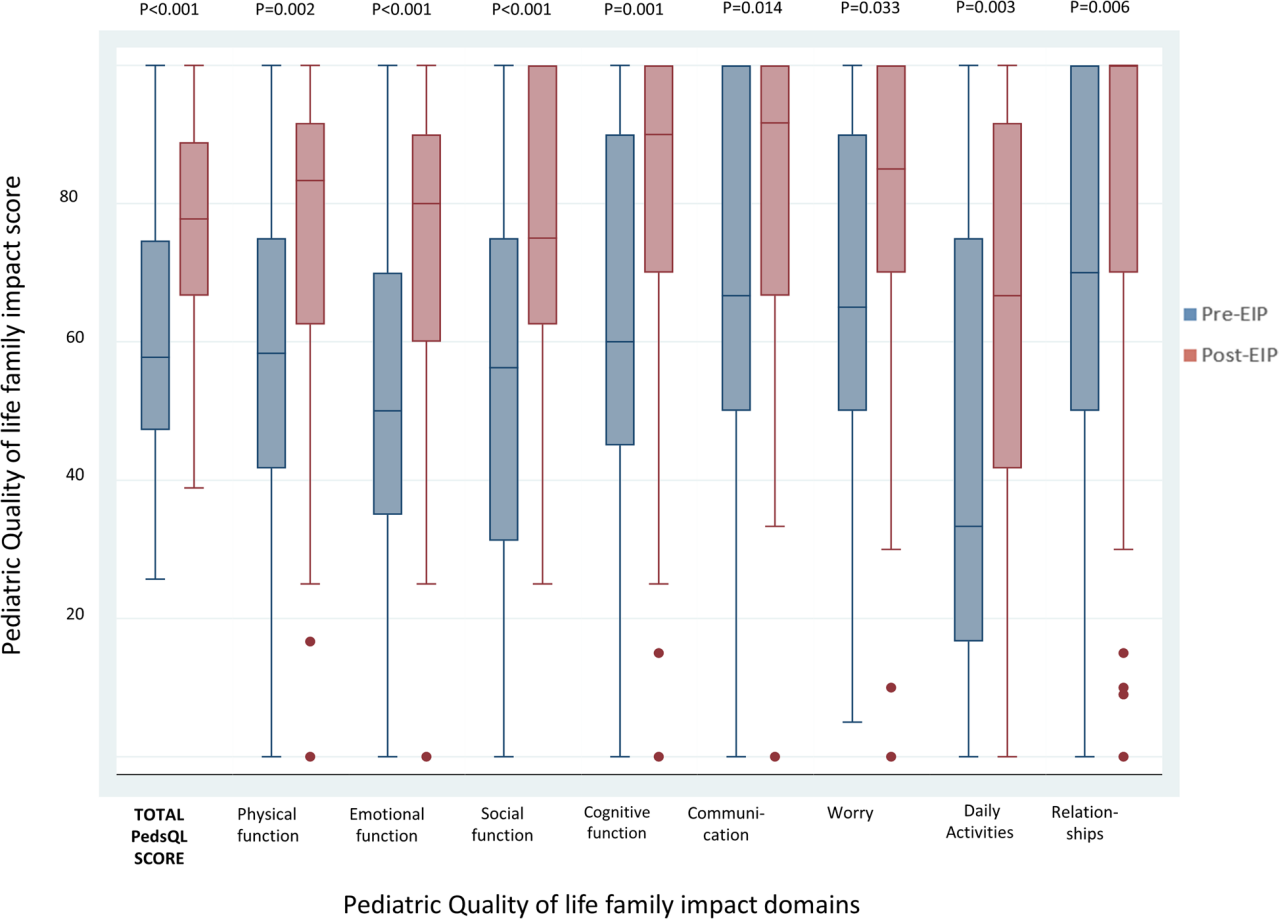
Total Pediatric Quality of Life family impact module scores and sub-scores for each domain, pre- and post- Early Intervention Programme (*n* = 48)

Comparison of quality of life and nutritional outcomes pre- and post-intervention are presented in Table 3 and Fig. 7. Overall, the median total PedsQL score increased by 21% (median difference + 12, IQR: + 4, + 27, *p* < 0.001) after receiving the programme (Table 3). The largest increase was seen in the sub-scores of emotional and social functioning, by 40 and 30% respectively (*p* < 0.001). Table 4 shows the comparison of quality of life and nutritional outcomes pre- and post-intervention when analysis was restricted to only those with satisfactory attendance (≥6 modules).

**Table 3:** Total Pediatric Quality of Life scores and sub-scores for each domain, and nutritional outcomes, pre- and post-Early Intervention Programme (*n* = 48)

Family quality of life	Median score (IQR) pre-EIP	Median score (IQR) post-EIP	Median change (IQR)	*p*-value^1^
TOTAL PedsQL score	57 (48, 75)	78 (65, 89)	12 (4, 27)	< 0.001
Sub-scores:				
Physical functioning	58 (43, 79)	83 (59, 92)	13 (0, 40)	0.002
Emotional Functioning	50 (31, 70)	80 (56, 94)	20 (0, 45)	< 0.001
Social functioning	63 (33, 75)	75 (63, 100)	19 (0, 47)	< 0.001
Cognitive functioning	65 (40, 90)	88 (70, 100)	15 (0, 40)	0.001
Communication	67 (50, 100)	88 (67, 100)	0 (−13, 42)	0.014
Worry	65 (56, 90)	83 (66, 100)	5 (−8, 25)	0.033
Daily activities	42 (10, 75)	67 (44, 92)	8 (− 4, 46)	0.003
Family relationships	80 (50, 100)	95 (70, 100)	10 (0, 33)	0.006
Nutritional outcomes	**Pre-EIP**^**2**^	**Post-EIP**^**3**^		
MUAC (cm), mean (range)	13 (9, 17)	13.7 (9, 19)		0.10^1^
MUAC < 12.5 cm, moderate- severe acute malnutrition	14 (35%)	7 (17%)		0.07^4^
MUAC < 11.5 cm, severe acute malnutrition n (%)	7 (18%)	5 (12%)		0.73^4^

*EIP* Early Intervention Programme, *PedsQL* Pediatric Quality of Life Inventory TM Score,Family Impact module 2.0, *IQR* Inter-quartile range, *MUAC* Mid-upper arm circumference^1^; From Wilcoxon signed rank test^2^; MUAC missing for 8 participants at baseline^3^; MUAC missing for 6 participants post-intervention; ^4^*p*-value from McNemar’s test

**Table 4:** Total Pediatric Quality of Life scores and sub-scores pre- and post-Early Intervention Programme for those attending ≥6 modules (*n* = 42)

Family quality of life	Median score (IQR) pre-EIP	Median score (IQR) post-EIP	Median change (IQR)	*p*-value^1^
TOTAL PedsQL score	54 (45, 74)	77 (65, 89)	15 (6, 32)	< 0.001
Sub-scores:				
Physical functioning	58 (41, 76)	83 (61, 89)	21 (0, 50)	< 0.001
Emotional Functioning	50 (30, 70)	80 (59, 91)	30 (5, 45)	< 0.001
Social functioning	63 (36, 75)	75 (63, 91)	19 (0, 38)	< 0.001
Cognitive functioning	53 (39, 90)	80 (65, 100)	20 (0, 40)	0.005
Communication	67 (42, 100)	83 (67, 100)	33 (−17, 42)	0.013
Worry	65 (50, 90)	83 (64, 100)	10 (0, 30)	0.042
Daily activities	33 (6, 67)	58 (40, 85)	8 (−8, 50)	0.001
Family relationships	70 (48, 93)	90 (68, 100)	15 (0, 30)	0.008

*EIP* Early Intervention Programme, *PedsQL* Pediatric Quality of Life Inventory TM Score, *IQR* Inter-quartile range

MUAC was measured in 83% (40/48) of children pre-EIP and 88% (42/48) post-EIP (total 9 children had missing MUAC at baseline or endline, of which 5 children were missing MUAC at both timepoints). At recruitment, 35% (14/40) were classified as having moderate-severe acute malnutrition with a MUAC of < 12.5 cm, compared to 17% (7/42) after completion of the EIP (*p* = 0.07, Table 3).

Caregivers and EIP facilitators reported in the discussions that the EIP had a positive impact on their children, themselves as caregivers, and the wider family. All caregivers reported improved knowledge on causes of disability and changed attitudes towards children with disabilities. One caregiver said, *“Before, I used to think CP was a curse and witchcraft but the training has given the understanding of what CP is and its cause.”* Another commented, *“It gave me confidence, I got to know what happened to my child and I appreciated the fact I was not alone.”* Most caregivers mentioned that their social, physical, and emotional health had improved. Participants reported that increased confidence in tackling stigma reduced stress and anxiety, and had a positive impact on their own physical health and on their child. One caregiver said, *“...the experience and interaction with other mothers and their CP children gives me confidence. I have been able to relate freely with others and it has reduced on my stigma.”* Caregivers also reported that improved knowledge positively impacted on caring for their child such as feeding safely with nutritious food; accepting, loving and encouraging their child; and advocating for their child in the face of discrimination had improved their child’s health, wellbeing and quality of life. One mother said, *“I appreciate the nutrition and feeding skills they gave us because I can see a change. He has put on some weight and my mother finds it easier to feed him because she has the skills now.”* The EIP also had a positive impact on the understanding of disability, and acceptance of children with disabilities among the wider family. One grandmother commented, *“The families’ thinking has now changed... I spend more time with him now and talk and sing to him while I work”,* while an aunt said, *“Our attitude has changed, we now love him and we help his mother with the exercises she has shared with us.”*

Caregivers felt supported to access services through the trained HCWs in their local communities. Prior to the EIP, caregivers complained about inadequate access to services. Both caregivers and EIP facilitators mentioned that the EIP provided basic training in home-based care and that local HCWs were better equipped with the knowledge and resources to manage their children effectively. Caregivers reported that due to improved local HCW knowledge, they no longer had to travel far to access specialised centres for their children as they could be treated at a local health centre.

## Discussion

In this secondary analysis we examined the feasibility, acceptability and impact of a programme of early detection and intervention for children with developmental disability, in a rural sub-Saharan African setting. This contributes to the important area of early intervention for children with developmental disabilities, for which data is still lacking particularly in LMICs. Our mixed-methods evaluation found early detection and intervention to be feasible and acceptable in this setting, and showed positive impacts on HCW knowledge, referral rates of affected children, and family quality of life. Several important programmatic barriers were identified including stigma, poverty, gender equality and geographical spread of enrolled families.

Early child development training and mentorship for HCWs was found to be feasible and acceptable with high attendance at initial training, and significant improvements in knowledge and confidence. However, only two-thirds of HCWs were able to return for the refresher training 6 months later. Difficulties in delivering ongoing training and supervision to HCWs is a common issue in ECD programming, with high staff turnover and poor retention cited as a challenge [24]. Commitment by district health services to reduce staff rotation may improve retention and facilitate retention of knowledge and skills. Despite challenges in sustainability of training, a significant increase in the annual referral rate for children with developmental disabilities was seen. The trend in referrals reduced several months after initial HCW training, which may have also been related to the onset of the rainy season in addition to attrition of trained HCWs at referring sites. Subsequent to refresher training in October, an increase in referrals for November was seen. The decrease in referrals to KCDC in December in both years could be explained by the annual 3-week closure of KCDC for the Christmas holiday. Whilst referrals were not always assessed using a validated neurodevelopmental tool due to time constraints, all children were clinically assessed by trained therapists and classified according to a recognised classification system [25]. Almost all referrals were found to be appropriate with the number of referred children without developmental disability reported as few by trained therapists. This is an important finding for the field, as there is no universally agreed referral threshold for early intervention programmes for children with developmental disabilities [26]. However, an international group is currently field testing recently developed population-level metrics (Global Scales for Early Development), and future plans include creating a global individual-level measure for screening [27].

Our community-based, participatory early intervention programme was found to be feasible and acceptable to facilitators (‘expert-parents’) and enrolled families, although notable barriers to access were identified including geographical spread of participants and poverty, making transport a substantial challenge for many families. Despite these challenges, over three-quarters of families had satisfactory attendance of 6 or more modules. Family engagement with the programme was high, with groups running at maximum capacity depending on the availability of facilitators and location of residence. Caregivers reported that they felt services were more accessible, an important benefit of the programme given the widespread paucity of services for children with disability in LMICs [28]. Whilst efforts were made to run groups as locally as possible to families, due to the large geographical spread they often still had to travel some distance. Creating a sustainable delivery platform with more geographically diverse facilitators, would increase access and attendance to the programme and facilitate a local network of peer support for families. However capacity for ongoing mentoring by trained therapists is an important consideration in scale-up to ensure maintenance of high fidelity programme delivery, as highlighted in previous literature [24].

Existing literature shows that caregivers of children with developmental disabilities report more negative experiences than caregivers of children without developmental disabilities, and that this is exacerbated in low-income settings [29]. This includes caregiver mental health problems such as depression and anxiety [30–33]. Previous work in Uganda found that mothers faced substantial social, emotional and financial difficulties, and stigma [2], which can lead to social exclusion [34]. A significant improvement in family quality of life was seen in the pre-post evaluation with the largest effects in social and emotional functioning. This was supported by caregivers reporting an increase in their knowledge, confidence and skills to care for their child, and a change in their attitudes and that of family members, reducing emotional stress and self-stigma. Improved community engagement has also been seen in earlier pilot work in the capital city of Kampala [19] and in the evaluation of a similar programme in Ghana [33]. Caregivers (the vast majority being mothers) reported that the programme empowered them to care for their child, access services, and tackle stigma. This empowerment has the potential to address gender inequality which can have wide-ranging positive impact on children and their families. In addition, the proportion of children with moderate-severe malnutrition reduced following completion of the intervention, which is important due to the widespread issue of poor nutrition in this population and the fact that development can be further impaired by this [34]. The reduction was not significant, although as nutritional status would commonly worsen over time in children with developmental disabilities, this still may represent positive impact. Caregivers also reported that feeding had improved after obtaining skills through the programme.

### Strengths and limitations

This study adds to the limited evidence base on intervention programmes for children with disability in low-resource settings. We utilised a mixed-methods approach to offer a more comprehensive evaluation, and the quantitative and qualitative findings supported each other to show positive impact in both early detection and intervention. Important programmatic barriers were identified including regular rotation of HCWs being a barrier to sustainability, and wide geographic spread and poverty as barrier to access for families in this context.

Our evaluation has some limitations. Firstly, interpretation of findings is limited by study design and a range of challenges in conducting rigorous monitoring and evaluation in this resource-limited setting. Pre−/ post-evaluation study designs are open to bias and lack a control group meaning findings may be biased or attributed to factors external to the intervention which must be considered when interpreting the positive study findings. The relatively small sample size, low number of participants with pre- and post-evaluation data and lack of a control group, may limit data interpretation. Evaluation data were on occasion collected by programme staff, which may have introduced bias and led to overestimation of impact. Whilst HCW confidence was self-assessed, pre- / post- assessments of knowledge were evaluated by those delivering the training which could have introduced observer bias. In addition, internal consistency was not assessed. However, encouragingly the qualitative findings, led by an independent social scientist not involved in programme delivery, mirrored the positive quantitative findings. Finally, findings may not be generalisable to other settings due to the rural context with high levels of poverty, however the results do mirror those reported during programme piloting in an urban setting [19].

## Conclusion

With a static global burden of child disability and lack of support services, there is a need for improved early detection and community-based early intervention programmes for children and their families, to optimise outcomes. A programme of early detection and early intervention were found to be feasible and acceptable in this rural African setting, and have positive impact on mixed-methods evaluation. HCW training on ECD improved HCW knowledge, skills, attitudes and practice, and the EIP improved family quality of life, including emotional stress and self-stigma. Barriers to scale-up identified included the wide geographical area, high levels of poverty, and social stigma. Whilst this study had limitations due to challenges in collecting reliable data in a resource-limited setting, our findings demonstrate the value of a mixed-methods approach for evaluating complex interventions, and adds to the limited evidence base in LMICs regarding early intervention programmes for children with disability. Further research into the implementation of such services in LMICs on a larger scale is urgently needed.

## Data Availability

The datasets generated and analysed during the current study are not publicly available, as informed consent was not explicitly obtained from participants for this. However they are available from the corresponding author on reasonable request.

## Abbreviations

ECD: Early Child Development
EIP: Early Intervention Programme
FGD: Focus Group Discussion
HCW: Healthcare Worker
KCDC: Kyaninga Child Development Centre
LMIC: Low- and Middle-Income Country
LSHTM: London School of Hygiene & Tropical Medicine
MRC/UVRI: Medical Research Council/ Uganda Virus Research Institute
MUAC: Mid-Upper Arm Circumference
PedsQL: Pediatric Quality of Life Inventory TM
